# Evaluation and comparison of different breast cancer prognosis scores based on gene expression data

**DOI:** 10.1186/s13058-023-01612-9

**Published:** 2023-02-08

**Authors:** Avirup Chowdhury, Paul D. Pharoah, Oscar M. Rueda

**Affiliations:** 1 Centre for Cancer Genetic Epidemiology, University of Cambridge, Cambridge, UK; 2 MRC Biostatistics Unit, University of Cambridge, East Forvie Building, Forvie Site, Robinson Way, Cambridge Biomedical Campus, Cambridge, CB2 0SR UK

**Keywords:** PREDICT, Breast cancer, Prognosis, Genomic score, Chemotherapy, Calibration, Discrimination, Reclassification

## Abstract

**Background:**

Breast cancer is one of the three most common cancers worldwide and is the most common malignancy in women. Treatment approaches for breast cancer are diverse and varied. Clinicians must balance risks and benefits when deciding treatments, and models have been developed to support this decision-making. Genomic risk scores (GRSs) may offer greater clinical value than standard clinicopathological models, but there is limited evidence as to whether these models perform better than the current clinical standard of care.

**Methods:**

PREDICT and GRSs were adapted using data from the original papers. Univariable Cox proportional hazards models were produced with breast cancer-specific survival (BCSS) as the outcome. Independent predictors of BCSS were used to build multivariable models with PREDICT. Signatures which provided independent prognostic information in multivariable models were incorporated into the PREDICT algorithm and assessed for calibration, discrimination and reclassification.

**Results:**

EndoPredict, MammaPrint and Prosigna demonstrated prognostic power independent of PREDICT in multivariable models for ER-positive patients; no score predicted BCSS in ER-negative patients. Incorporating these models into PREDICT had only a modest impact upon calibration (with absolute improvements of 0.2–0.8%), discrimination (with no statistically significant *c*-index improvements) and reclassification (with 4–10% of patients being reclassified).

**Conclusion:**

Addition of GRSs to PREDICT had limited impact on model fit or treatment received. This analysis does not support widespread adoption of current GRSs based on our implementations of commercial products.

**Supplementary Information:**

The online version contains supplementary material available at 10.1186/s13058-023-01612-9.

## Introduction

Breast carcinoma is an unregulated growth of cells within the functional units of breast epithelium [1]. It is one of the three most common cancers worldwide (with an estimated 2.26 million new cases in 2020) and the most common malignancy in women [2, 3]. Treatment approaches for breast cancer are diverse and varied: in the UK between 2013 and 2014, 81% of breast cancer patients had surgery as part of their primary treatment regimen, 63% had radiotherapy, 34% had chemotherapy [4], and 62% had endocrine therapy [5].

Clinicians managing breast cancer must balance the risks of treatment against the potential benefits. Prognostic scores have been developed to assist in these predictions. These include clinical risk scores and genomic risk scores (GRSs). PREDICT is one example of a clinical score. It is based on a multivariable Cox proportional hazards model incorporating patient age, tumour size, tumour grade, tumour protein expression (ER, HER2 and KI67), positive nodes and mode of diagnosis [6, 7]. It provides estimates of absolute treatment benefit for hormone therapy, chemotherapy, adjuvant trastuzumab and bisphosphonate therapy. PREDICT is recommended by the National Institute for Health and Care Excellence (NICE) as a tool for supporting clinical decisions on adjuvant treatment benefit [8] and has been endorsed by the American Joint Committee on Cancer [9]. The underlying model is flexible, enabling additional biomarkers to be incorporated.

GRSs (also called genomic prognostic scores) based on RNA gene expression data were developed in response to the concern that clinicopathological features are imperfect estimators of disease risk and chemosensitivity [10]. They have the theoretical advantages of optimal use of continuous variables and added robustness (e.g. by gathering information on ER activity through a cluster of genes) [11]. Many GRSs have been developed, but few are commercially available and fewer still are endorsed by clinical bodies. At present, only Oncotype DX [12], EPClin [13] and Prosigna [14] have been approved for use in clinical practice in the UK under specific circumstances [15]. Another signature, MammaPrint [16], was deemed clinically effective but not cost-effective.

Several key metrics are used to assess model fit, including calibration, discrimination and reclassification. Calibration is defined as the agreement between observed outcomes and predictions, often presented as an absolute difference in values. Discrimination is a model’s ability to differentiate between those with and without the outcome [17], typically expressed using *c*-indices [18]. Reclassification refers to the movement of individuals between risk categories with the introduction of a new prediction model (or extension of a model through the addition of new variables) [17, 19]. Even if the calibration or discrimination of a model does not change, a change in risk categories may result in an individual receiving different treatment according to clinical guidelines [19].

There is a paucity of evidence comparing GRSs to current validated clinical scores. Previous studies comparing GRSs to clinicopathological scores use models less comprehensive than PREDICT and tend to reduce continuous variables into categories [20–28].

This study aims to assess whether GRSs provide any additional clinical benefit beyond PREDICT, the current standard of care, using a head-to-head comparison in an external cohort. It also analyses the impact of model fit when GRSs are incorporated into the PREDICT algorithm. GRSs included for comparison were those referred to by NICE in their most recent guideline [15]: EPClin, Oncotype DX, Prosigna and MammaPrint.

## Material and methods

Linked clinical and gene expression data were obtained from the METABRIC study [29, 30], described in detail elsewhere. The hazard ratio (HR) functions from PREDICT version 2.1 were used in this analysis [7]. Surrogate KI67 status was calculated using gene expression data for *MKI67*, the gene which codes the KI67 protein, using the *mclust* package [31]. Proportions of KI67 status grouped by cancer grade, stage, number of lymph nodes positive and hormonal status were similar to those previously reported [32].

Four GRSs were adapted in line with the specifications of their respective papers—EndoPredict, Oncotype DX (ODx), Prosigna and MammaPrint. Code was adapted from the *genefu* R package [33] to make it suitable for use on z-score normalised expression data. 10-year breast cancer-specific survival (BCSS) was the outcome of interest, defined as the percentage of patients who did not die from breast cancer over ten years.

### Building Cox proportional hazards models

Cox proportional hazards models were built using the *survival* package [34]. The primary outcome of interest was breast cancer-specific death. Separate models were built for ER-positive and ER-negative patients, since the baseline hazard is different in these two groups [7, 12]. In all models, the PREDICT prognostic index was constrained to have a coefficient of one and it was included as an offset. This avoids overfitting and serves as an independent validation of the PREDICT model. No constraints were placed upon GRSs as this information was unavailable, and so this does not serve as an independent validation of these scores as used clinically. Unlike in the original PREDICT model, follow-up was not censored at 15 years.

Univariable models were built for each GRS in turn. Multivariable models were built using PREDICT plus a GRS to assess whether the prognostic information provided by GRSs was independent of PREDICT. Since PREDICT is already a validated multivariable model which incorporates key clinical factors known to be associated with breast cancer prognosis, no additional terms were included in the model to prevent overfitting; for this reason, EndoPredict was used in place of EPClin, and the ROR-C score chosen for Prosigna, in multivariable models. Models were built using single GRSs, since multiple scores are unlikely to be used simultaneously in a clinical setting due to prohibitive cost.

### Adding GRS terms into the PREDICT algorithm

We also assessed the impact of including GRSs on the calibration, discrimination and reclassification of the PREDICT algorithm. GRS terms were incorporated as additional terms into PREDICT after rescaling such that the average HR across the GRS distribution was one. This ensures that the baseline hazard used in PREDICT is appropriate.

To account for differences in follow-up time, the expected 10-year survival probability of each patient was calculated using PREDICT. Calibration was reported as the absolute difference in 10-year BCSS between the predicted results (the mean survival of all patients as reported by each algorithm) against the observed results (calculated using the *survival* package [34]). Discrimination was reported for each algorithm in turn by producing a univariable Cox proportional hazards model, and statistical significance tested using the *survcomp* [35] package. Goodness of fit was reported using log-likelihoods and tested using one-way ANOVA tests. Log-likelihoods are equivalent to Akaike information criterion in this case since all models have the same number of variables.

In order to account for the effect of using the same observations to estimate the hazard ratios and to measure model performance, we computed the optimism [36] using a bootstrap procedure adapted from the rms R package [36]. We resampled 100 times from the original dataset, fitted the model for each of these samples and compared the performance estimated in the bootstrap sample with a testing sample that contained the observations not sampled in each iteration. The difference between them is the optimism and gives an indication of the amount of overfitting in the model.

The effect of second-generation chemotherapy upon BCSS was used to assess reclassification. Locally, the Cambridge Breast Unit uses PREDICT to stratify patients into three groups according to the predicted benefit of adjuvant chemotherapy: absolute increases in BCSS of < 3%, 3–5% and > 5% [37]. The first group is usually not offered chemotherapy, and chemotherapy is recommended in the third group; for the middle group, a discussion of the pros and cons of treatment is conducted. These thresholds were used to categorise patients; reclassification was assessed using reclassification tables.

All analyses were conducted in RStudio (version 4.1.0, RStudio, Inc., MA, USA); analysis code and data are provided as Additional file 1.

## Results

### Study population characteristics

Matched clinical outcome and gene expression data were available for 1980 patients in the METABRIC cohort (Table 1). Median follow-up in the study population was 9.56 years (range 0–29.2 years). There were 646 breast cancer-specific deaths during the study period.

**Table 1:** Study population characteristics

Characteristic	Patients (*n* = 1980)
Median age at diagnosis, years (range)	61.8 (21.9–96.3)
Inferred menopausal state (%)	
Pre-menopausal	424 (21)
Menopausal	1556 (79)
Oestrogen receptor status (%)	
Positive	1506 (76)
Negative	474 (24)
Progesterone receptor status (%)	
Positive	1040 (53)
Negative	940 (47)
HER2 receptor status (%)	
Positive	247 (12)
Negative	1733 (88)
Stage (%)	
In situ	12 (1)
I	501 (25)
II	825 (42)
III	118 (6)
IV	10 (1)
Not specified	514 (26)
Grade (%)	
1	169 (9)
2	772 (39)
3	955 (48)
Not specified	84 (4)
Median tumour size, mm (range)	23 (1–403)
Lymph node involvement (%)	
Negative	1043 (53)
Positive	937 (47)
Surgery (%)	
Breast-conserving	785 (40)
Mastectomy	1170 (59)
Not specified	25 (1)
Chemotherapy (%)	
None	1568 (79)
Second generation	395 (20)
Third generation	17 (1)
Radiotherapy (%)	
Not received	810 (41)
Received	1170 (59)
Hormone therapy (%)	
Not received	764 (39)
Received	1216 (61)

### Cox proportional hazards models

Additional file 2: Table S1 summarises key metrics from univariable Cox proportional hazards models. In the ER-positive cohort, all scores except MammaPrint had statistically significant HRs. No GRS had a significant HR in ER-negative patients.

The discrimination of PREDICT (c-index 0.687) was better than GRSs in ER-positive cases; MammaPrint was the best GRS (0.652). GRS discrimination was poor for ER-negative patients with PREDICT performing substantially better (0.667).

In multivariable models, EndoPredict and MammaPrint statistically significantly improved the fit of PREDICT in ER-positive patients (Table 2). While adding Prosigna significantly improved model fit in ER-negative patients, the overall hazard ratio remained non-significant. There was no significant change in discrimination with the addition of GRSs in either ER-positive or ER-negative patients.

**Table 2:** Multivariable Cox proportional hazards models for each genomic prognostic signature adjusted for PREDICT in (a) ER-positive and (b) ER-negative patients

Model	Hazard ratio (95% CI)	Log-likelihood	Model fit *p* value	c-index (95%CI)	c-index optimism
(a)					
PREDICT	2.72^†^ (NA)	− 2860.6	–	0.687 (0.661–0.713)	–
Oncotype DX	1 (0.99–1)	− 2859.1	0.0835	0.693 (0.667–0.718)	− 4.17 × 10^–4^
EndoPredict	1.13 (1.02–1.24)	− 2858	0.024	0.692 (0.666–0.717)	0.003
MammaPrint	0.52 (0.17– 0.87)	− 2853.9	2.32 × 10^–4^	0.699 (0.674–0.724)	6.72 × 10^–4^
Prosigna	1.58 (1.11– 2.05)	− 2858.7	0.0546	0.694 (0.668–0.719)	− 8.68 × 10^–4^
(b)					
PREDICT	2.72^†^ (NA)	− 1072	–	0.667 (0.630–0.704)	–
Oncotype DX	0.998 (0.986– 1.01)	− 1072	0.77	0.667 (0.63–0.704)	0.011
EndoPredict	0.884 (0.657– 1.11)	− 1071.4	0.287	0.667 (0.630–0.705)	0.007
MammaPrint	0.854 (0.196– 1.51)	− 1071.9	0.638	0.669 (0.632–0.706)	0.005
Prosigna	0.252 (− 0.875–1.38)	− 1069.3	0.0166	0.661 (0.622–0.699)	7.84 × 10^–4^

95% CI, 95% confidence interval

^†^Hazard ratio has been constrained to this value

### Modified PREDICT algorithm

The PREDICT algorithm was modified to incorporate GRS coefficients from the multivariable models. All modified algorithms underestimated 10-year absolute survival in the METABRIC cohort (Table 3). Survival in the METABRIC cohort at 10 years was 74.0% for ER-positive patients and 58.5% in ER-negative patients. Estimates in ER-positive patients underestimated survival by between 12.2% and 13%, with the closest estimate from PREDICT + MammaPrint. Estimates in ER-negative patients underestimated survival by between 2.3 and 8.8%, with the closest estimate from PREDICT + Prosigna. Subgroup analyses are reported in Additional file 2: Figures S1–S6.

**Table 3:** Calibration of original and modified PREDICT models in the METABRIC cohort for (a) ER-positive and (b) ER-negative patients

Model	10-Year survival %	Difference	Difference optimism
(a)			
** METABRIC cohort**	**74.0**	Reference	Reference
PREDICT	61.1	− 13	–
PREDICT + Oncotype DX	61.3	− 12.8	− 0.694
PREDICT + EndoPredict	61.6	− 12.5	− 0.711
PREDICT + MammaPrint	61.9	− 12.2	− 0.640
PREDICT + Prosigna	61.2	− 12.8	− 0.706
(b)			
**METABRIC cohort**	**58.5**	Reference	Reference
PREDICT	49.7	− 8.8	–
PREDICT + Oncotype DX	50.5	− 8	− 0.307
PREDICT + EndoPredict	52.9	− 5.6	− 0.403
PREDICT + MammaPrint	48.3	− 10.2	− 0.150
PREDICT + Prosigna	56.2	− 2.3	− 0.336

In bold, reference survival (METABRIC cohort)

Including GRSs in PREDICT resulted in statistically significant improvements in model fit. Point estimates of discrimination were improved in ER-positive patients with the inclusion of any GRS. However, none of these changes were statistically significant.

The majority of patients remained in the same clinical group when using the original and modified forms of the PREDICT model. A total of 1878 patients were included in these analyses, with the remaining 102 excluded due to missing event data. There were 74 (4%) reclassifications when Oncotype DX was included in PREDICT. This was lower than those for EndoPredict (132; 7%), MammaPrint (154; 8%) and Prosigna (183; 10%).

The most important clinical category of ER-positive patients to consider is intermediate benefit, since the benefit of adjuvant chemotherapy is unclear in this group. Reclassification varied by GRS (Table 4). Similar numbers of patients were reclassified into and out of the intermediate benefit category with Oncotype DX (40 vs. 34) and EndoPredict (71 vs. 61). More patients were reclassified out of intermediate benefit than into it using MammaPrint (66 vs. 88), while more were classified as intermediate when using Prosigna (102 vs. 80).

**Table 4:** Reclassification tables comparing clinical categories of chemotherapy benefit from standalone PREDICT and PREDICT with (a) Oncotype DX, (b) EndoPredict, (c) MammaPrint and (d) Prosigna in ER-positive patients

	PREDICT + Oncotype DX		
	Low	Intermediate	High
(a)			
PREDICT			
Low	893	19	0
Intermediate	27	434	7
High	0	21	477

	PREDICT + EndoPredict		
	Low	Intermediate	High
(b)			
PREDICT			
Low	890	22	0
Intermediate	46	407	15
High	0	49	449

	PREDICT + MammaPrint		
	Low	Intermediate	High
(c)			
PREDICT			
Low	873	39	0
Intermediate	54	380	34
High	0	27	471

	PREDICT + Prosigna		
	Low	Intermediate	High
(d)			
PREDICT			
Low	877	34	1
Intermediate	60	388	20
High	0	68	430

No patients were reclassified from high to low benefit or vice versa when Oncotype DX, EndoPredict or MammaPrint were used; with Prosigna, 1 patient was reclassified from low to high benefit.

## Discussion

Overall, EndoPredict, MammaPrint and Prosigna demonstrated prognostic power independent of PREDICT in multivariable models in ER-positive patients; however, discrimination was not significantly improved. Incorporating GRSs into the PREDICT algorithm did not improve calibration, with underestimation of 10-year BCSS in the METABRIC cohort in ER-positive and ER-negative cohorts. Measures of discrimination were not significantly changed. GRS inclusion caused 4–10% of patients to be reclassified into different clinical categories.

This analysis addresses some key gaps in the current evidence base. In their most recent guideline on the topic, NICE [15] states that “there are no data available to compare the tumour profiling tests with PREDICT, or to define the clinical risk groups using PREDICT”. Previous studies tended to compare GRS against one another or against suboptimal clinicopathological parameters. This study used the current clinical standard of care, making it easier to assess whether clinical predictions are improved.

By creating modified versions of the PREDICT algorithm which accepted GRSs as an additional term, this analysis leveraged absolute risk predictions from PREDICT to allow inferences about the impact of GRSs on model fit. Although this is not the same as calculating the calibration of the scores themselves, it nonetheless demonstrates what the impact of combining PREDICT and GRSs would be.

PREDICT had poor calibration in this analysis, underestimating 10-year BCSS by 13%. Independent validation of PREDICT by Gray et al. [38] on the Scottish Cancer Registry showed much higher calibration, with 5% overestimation of 5-year mortality and 2% underestimation of 10-year mortality. The differences in findings between this analysis and previous work may be due to differences in the outcome of interest (overall survival versus BCSS) or the cohorts themselves (as outcomes in the general population may be different to those in a highly selected cohort like METABRIC, from the UK and Canada). It may also be due to calibration drift, where risk estimates change over time due to changes in population characteristics or disease incidence [39]. This may also explain the lower underestimates of calibration in ER-negative disease (Additional file 2: Figure S1), for whom treatment advances have been more limited.

There is a need to develop prognostic molecular scores which take into account the standard clinicopathological variables already used in a clinical setting and then explore how much additional benefit is gained from the inclusion of genomic signatures. In this way, the developed scores will reflect real-world clinical practice and be more relevant to clinical decision-making.

### Limitations and future work

A key limitation of this work is that surrogate GRSs were used rather than their commercial counterparts due to cost considerations. Although all GRSs in this study were derived from published papers in line with the original authors’ instructions, there is nonetheless a risk that these differ from commercial scores. Future work should use commercial scores where possible.

Due to the time period in which METABRIC patients were recruited, certain treatments (e.g. bisphosphonates) were less commonly used. There is also a risk of time-varying confounding due to available treatment regimens changing over time (and affecting patient survival). This is likely given that breast cancer survival rates have improved dramatically over the past decades, a finding attributed in major part to improved treatment [1, 2].

The use of pre-processed METABRIC data presented some challenges for conversion of GRSs. Changing the type of data normalisation post hoc is challenging and risks introducing further biases [40]. These issues continue to exist even if raw data are analysed; the only way to eliminate them is to use the genomic test with the official platform. This was not feasible due to the high cost of requesting such testing.

Only BCSS was considered in this analysis. Clinical outcome measures used in cancer are diverse and include measures of survival, disease recurrence and disease-free survival. Although these outcomes are correlated to some extent, future studies would benefit from considering multiple clinical outcomes to ensure that these findings are consistent and sustained across clinically relevant subgroups.

This study likely underestimates PREDICT’s performance, since several variables required for optimal prediction (screening status, KI67 status and bisphosphonate use) were unavailable. Although alternative methods were used to infer KI67 status, these are unlikely to be as accurate as established histopathological techniques.

Similarly, this analysis likely overestimates GRS prognostic power, since GRS coefficients were unconstrained, while PREDICT was constrained to one. This has the effect of allowing GRS coefficients to be re-estimated in the current dataset, effectively creating an overfitting problem whereby the predictive power of included variables is overestimated. The impact of this may be quite large: when univariable PREDICT models were built using the unconstrained variable, model fit was dramatically improved compared to the constrained model (log-likelihood -2823.6 versus –2860.2).

Although this study found that there were largely non-significant changes in model performance as a result of incorporation of genomic prognostic scores, these changes need to be modelled economically. In particular, the small number of reclassifications may be important at a health system level if they result in improvements in patient care (which reduce long-term costs of readmission, for example).

## Conclusion

This study evaluates the impact of adding GRSs to current standards of care for breast cancer predictive modelling using key model performance metrics (calibration, discrimination and reclassification). Three GRSs (EndoPredict, MammaPrint and Prosigna) demonstrated power to predict BCSS in breast cancer independent of PREDICT. However, incorporating these models into PREDICT had only a modest impact upon calibration (underestimating 10-year BCSS by around 12%), discrimination (with *c*-indices non-significantly different to the original PREDICT algorithm) and reclassification (with 4–10% of patients reclassified into different clinical categories). Performance was much better in ER-positive than ER-negative patients. Although these small improvements in model fit might be clinically useful, economic analyses are needed to assess whether this justifies the increased cost.

## Supplementary Information

**Additional file 1.** Code and data used in the study.

**Additional file 2.** Supplementary Tables and Figures.

## Data Availability

All data generated or analysed during this study are included in this published article (and its supplementary information files).
