# Supplementary material for: Evaluation and comparison of different breast cancer prognosis scores based on gene expression data
Source: Breast Cancer Res. 2023 Feb 8;25:17. doi: 10.1186/s13058-023-01612-9 (PMC9906838; doi:10.1186/s13058-023-01612-9)

**Table S1: Features of univariable Cox proportional hazards models of breast cancer-specific survival in (a) ER-positive and (b) ER-negative patients.** 95%CI: 95% confidence interval. ^†^Hazard ratio has been constrained to this value.

| (a) | **Model** | **Hazard ratio (95%CI)** | **Log-likelihood** | **c-index (95%CI)** | **c-index optimism** |
| --- | --- | --- | --- | --- | --- |
|  | PREDICT | 2.72^†^ | -2860.6 | 0.687 (0.661-0.713) | - |
|  | Oncotype DX | 1.02 (1.02-1.02) | -2877.3 | 0.642 (0.616-0.668) | -0.002 |
|  | EndoPredict | 1.51 (1.40-1.62) | -2891 | 0.614 (0.584-0.643) | -0.005 |
|  | MammaPrint | 0.18 (-0.16-0.52) | -2869.4 | 0.652 (0.624-0.68) | 1.6 x 10^-4^ |
|  | Prosigna | 8.59 (8.12-9.06) | -2874.6 | 0.647 (0.62-0.673) | -0.001 |
| (b) |  |  |  |  |  |
|  | PREDICT | 2.72^†^ | -1072 | 0.667 (0.63-0.704) | - |
|  | Oncotype DX | 0.99 (0.98-1) | -1082.6 | 0.514 (0.474-0.555) | 0.017 |
|  | EndoPredict | 0.85 (0.64-1.06) | -1082.1 | 0.529 (0.486-0.572) | 0.011 |
|  | MammaPrint | 1.26 (0.65-1.87) | -1082.9 | 0.513 (0.472-0.554) | 0.022 |
|  | Prosigna | 1.02 (-0.13-2.17) | -1083.2 | 0.508 (0.467-0.55) | 0.029 |

**Figure S1: 10-year breast cancer-specific survival predicted and observed in (a) ER-positive and (b) ER-negative patients.**


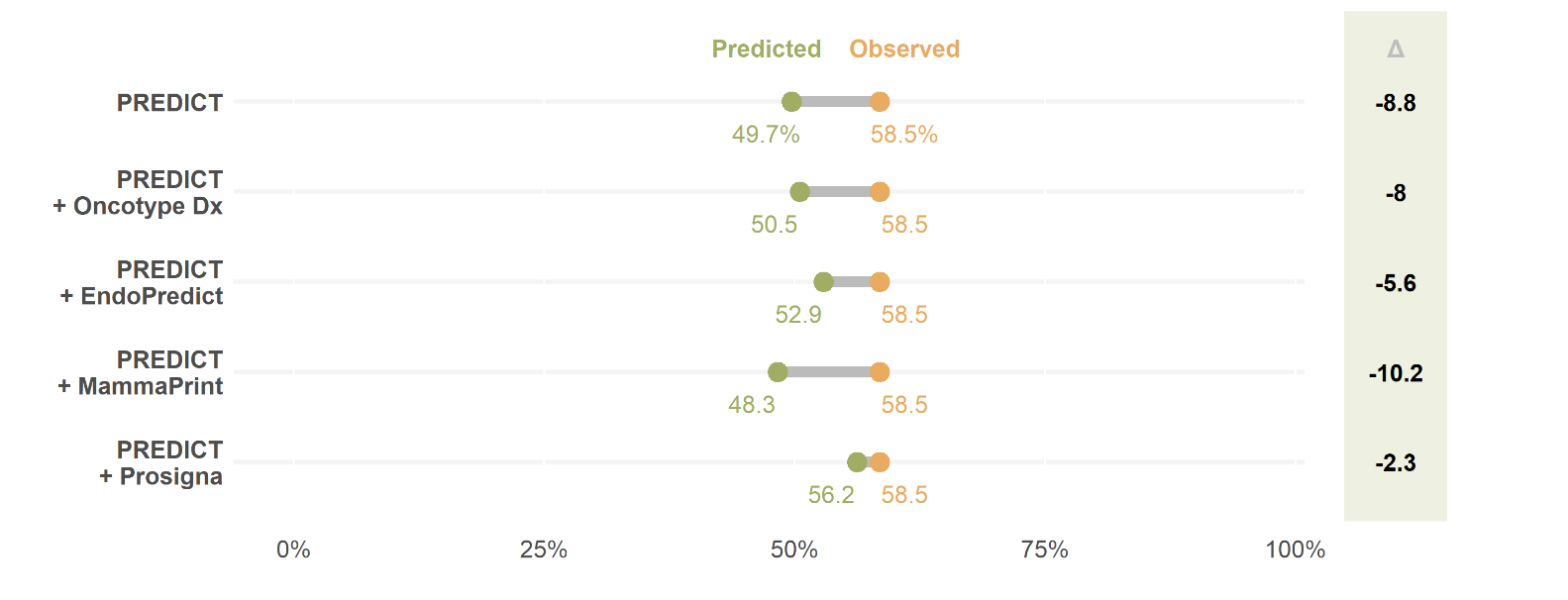

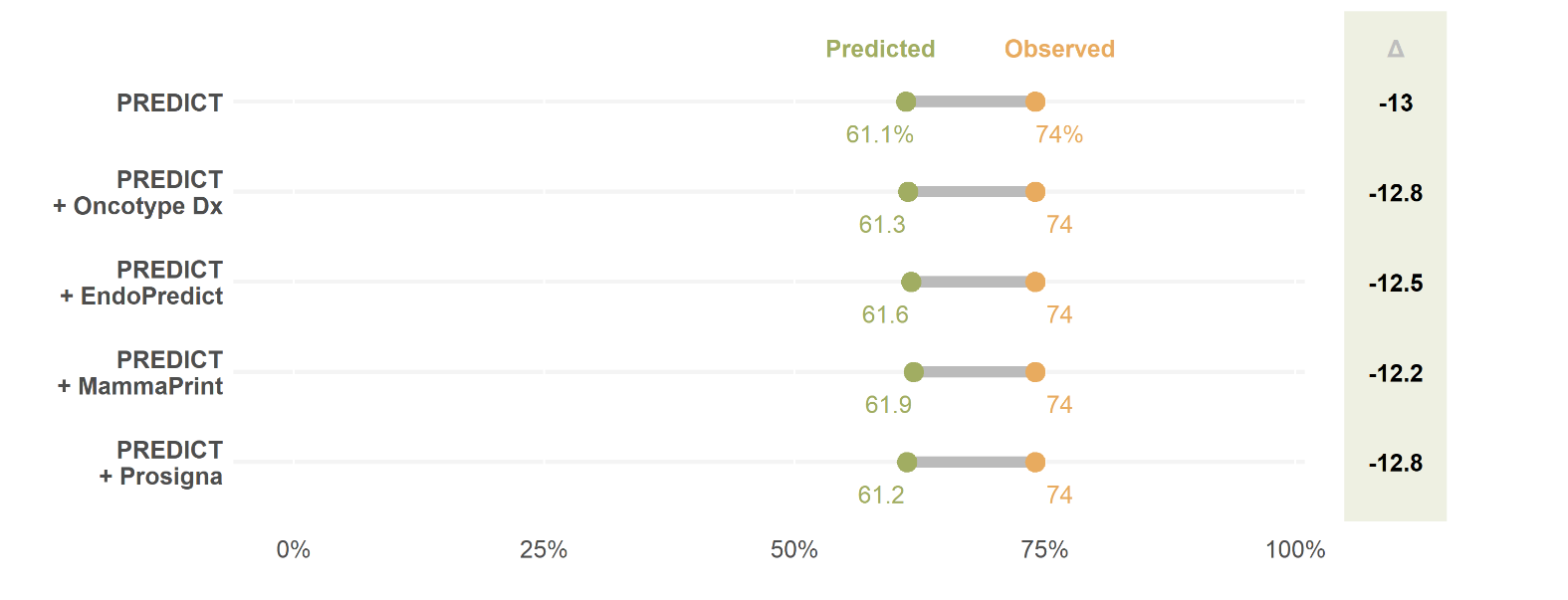


**(b)**

**(a)**

**Figure S2: 10-year breast cancer-specific survival predicted and observed in (a) ER-positive, node-negative, (b) ER-positive, node-positive, (c) ER-negative, node-negative and (d) ER-negative, node-positive patients.**


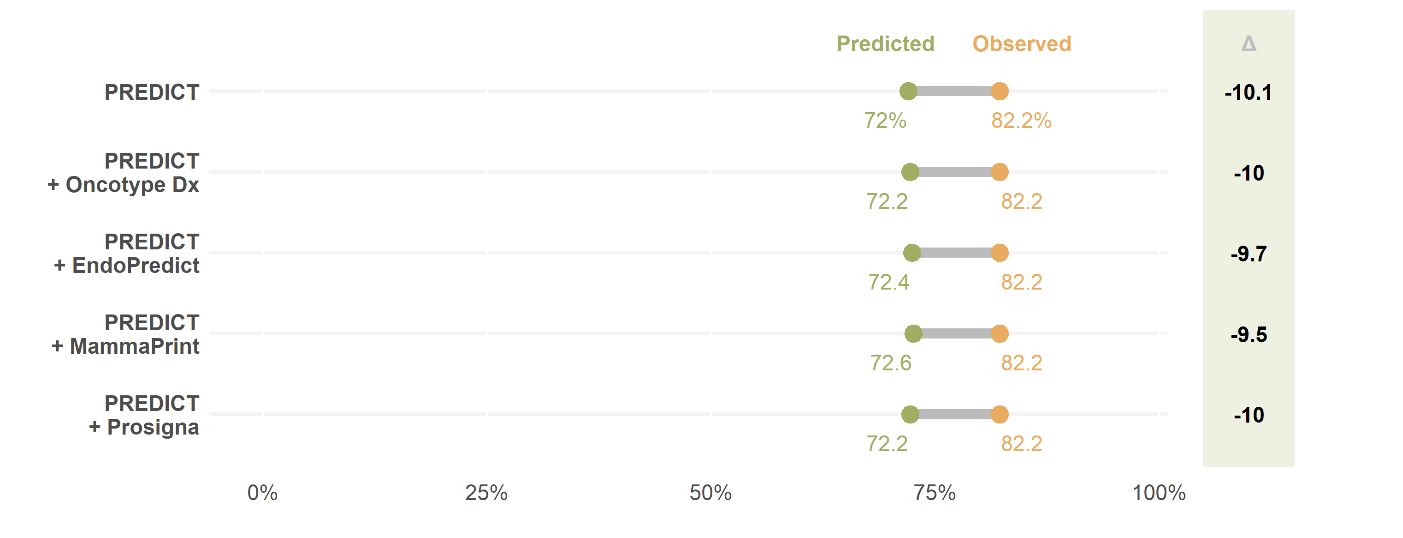

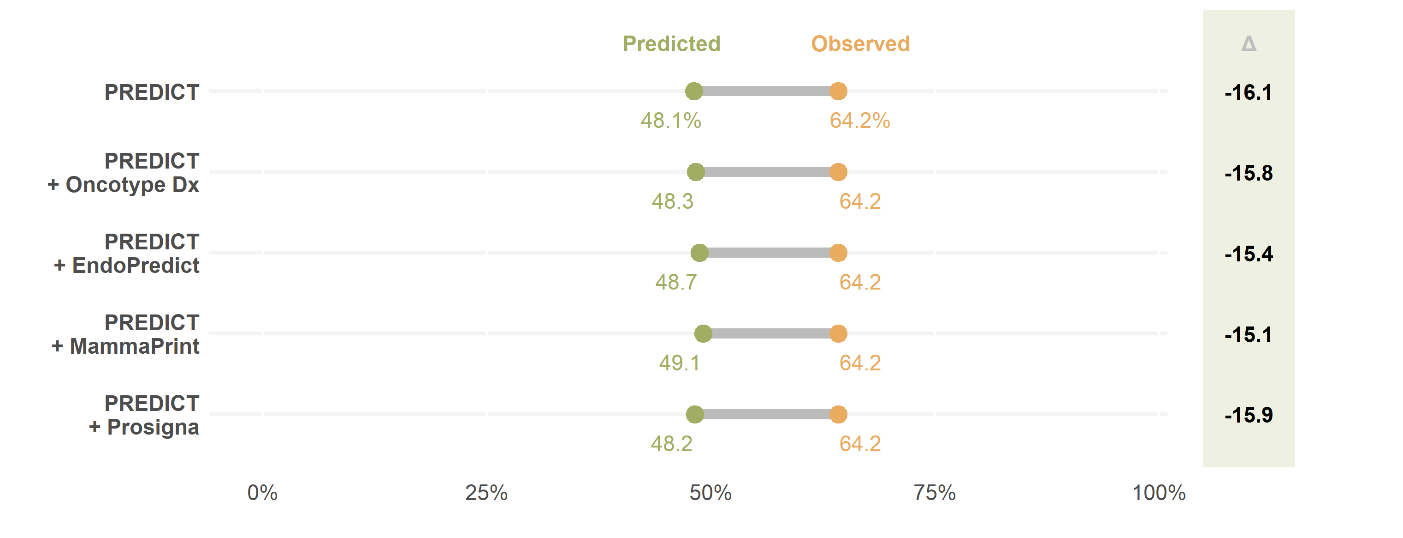

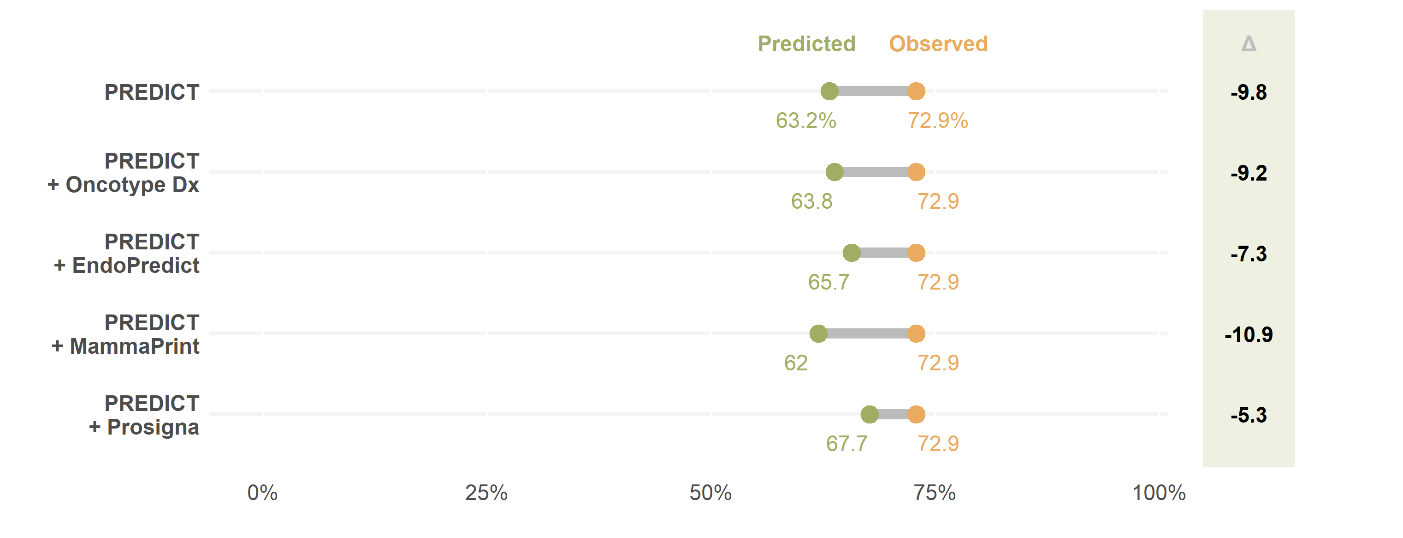

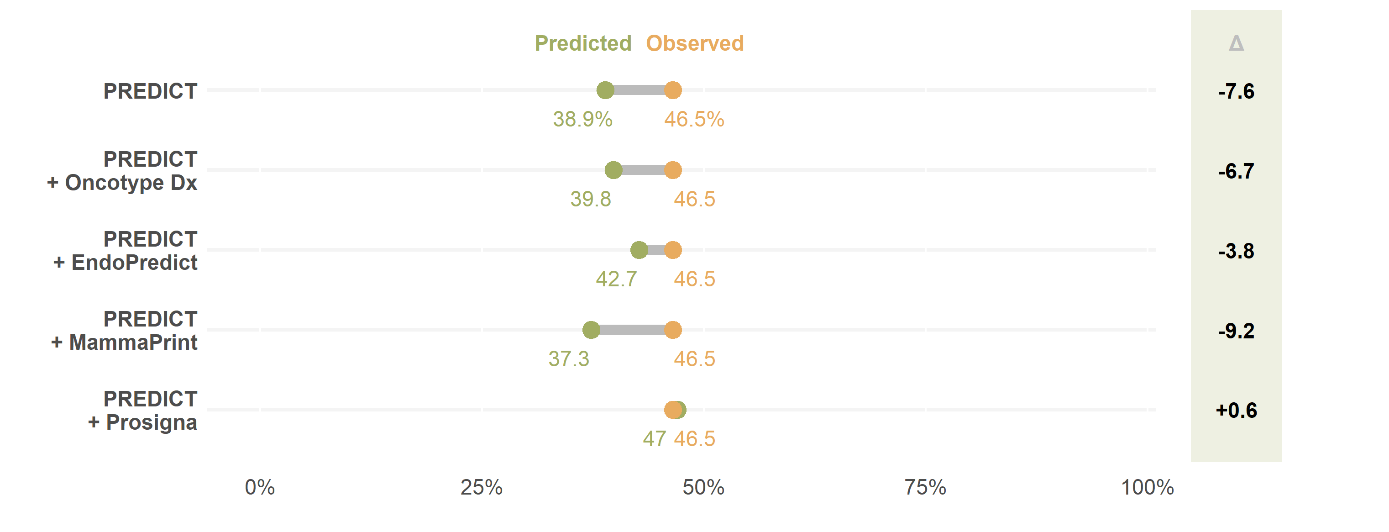


**(a)**

**(b)**

**(c)**

**(d)**

**Figure S3: 10-year breast cancer-specific survival predicted and observed in patients with (a) ER-positive, grade 1, (b) ER-positive, grade 2, (c) ER-positive, grade 3, (d) ER-negative, grade 1, (e) ER-negative, grade 2, (f) ER-negative, grade 3 disease.**


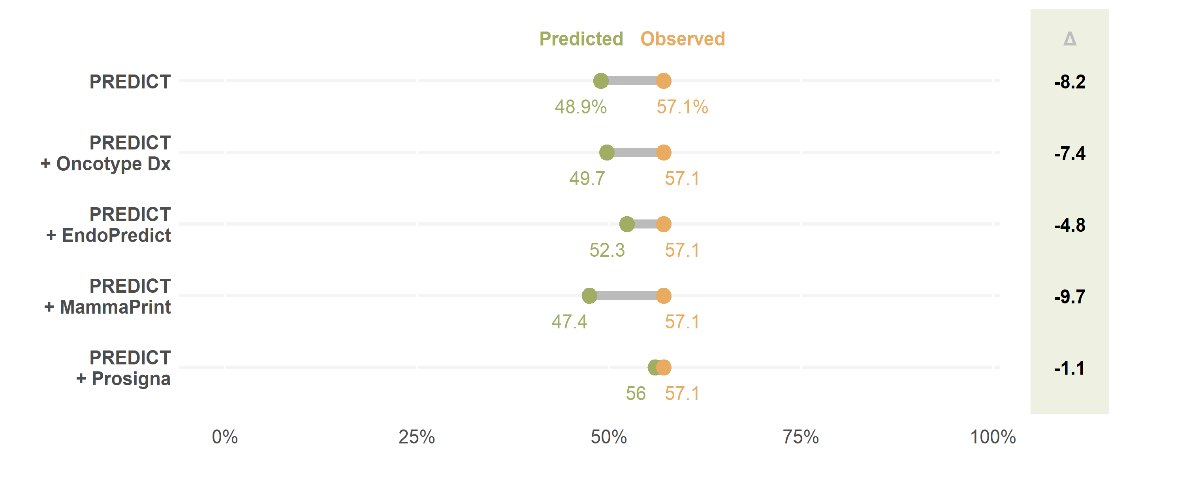

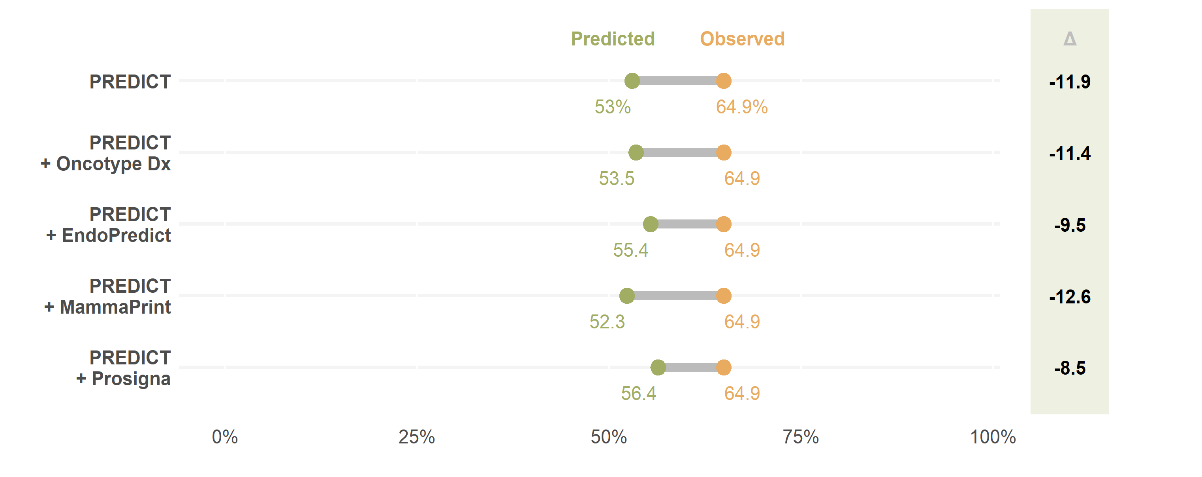

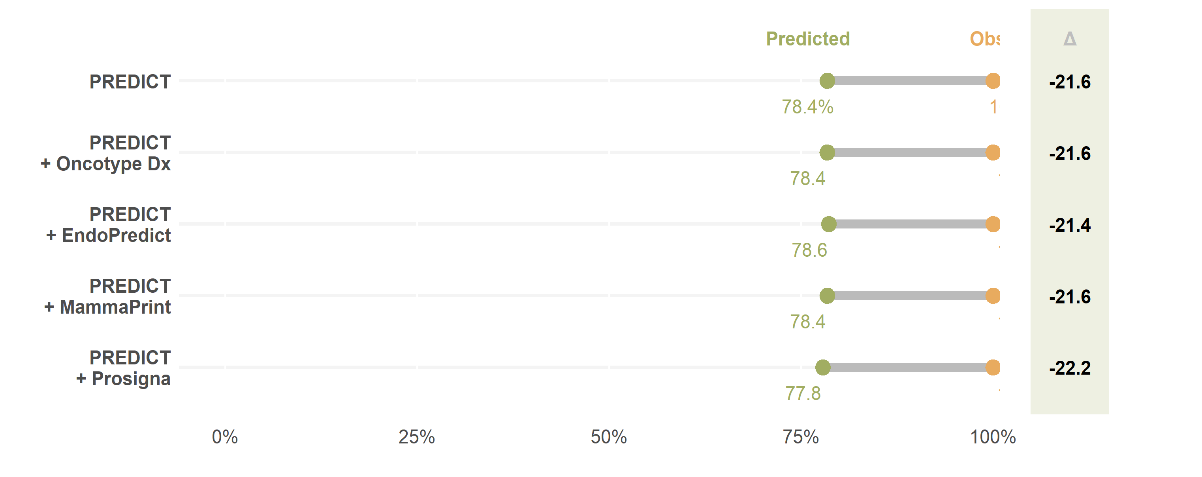


**(d)**

**(e)**

**(f)**


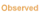

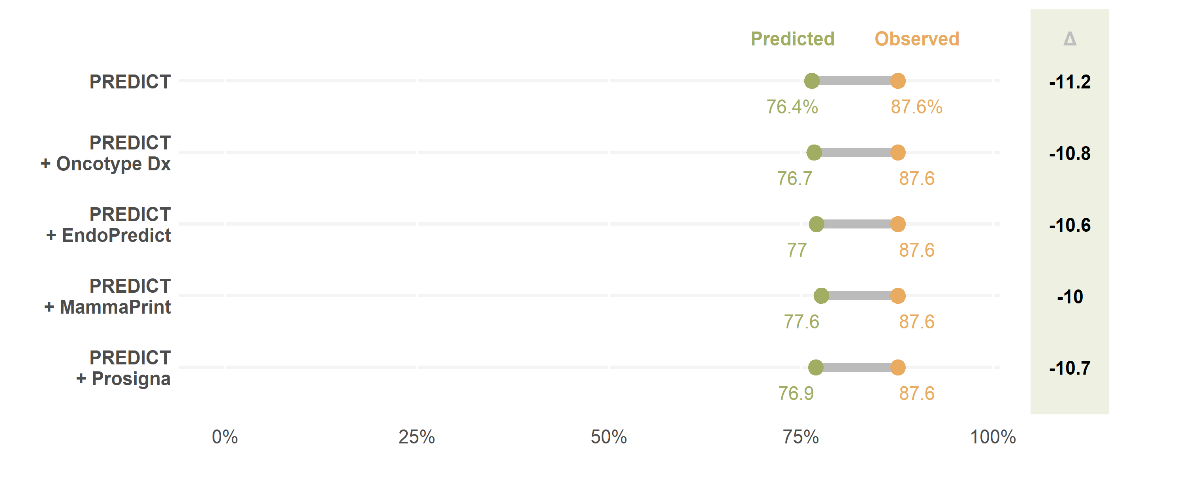

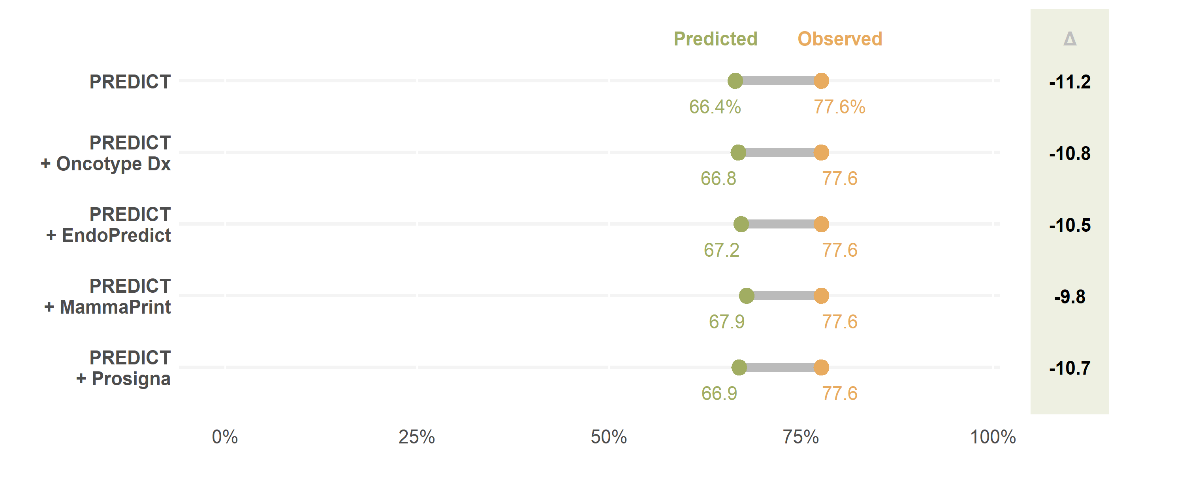

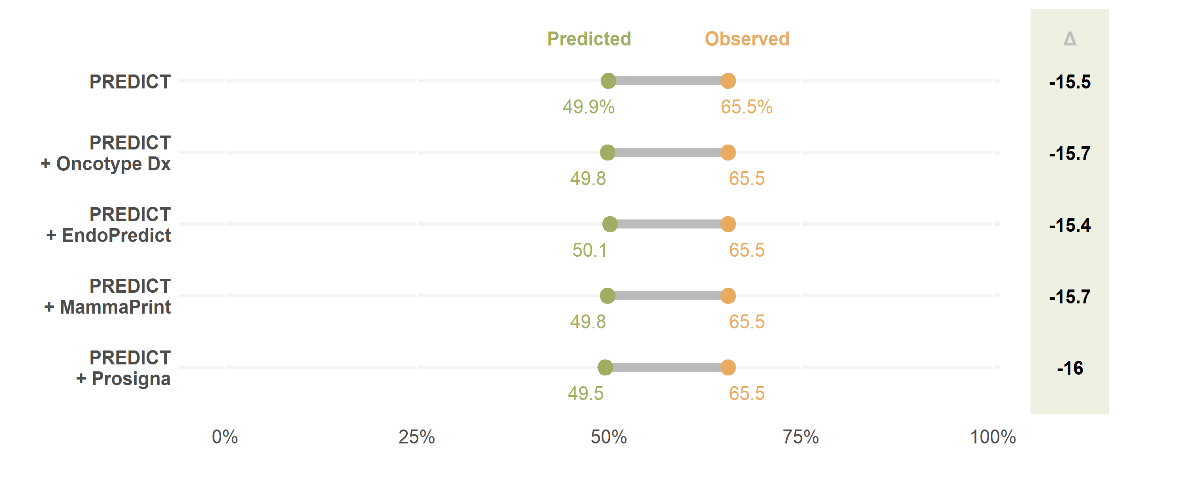


**(a)**

**(b)**

**(c)**

**Figure S4: 10-year breast cancer-specific survival predicted and observed in patients with ER-positive disease diagnosed in the (a) 1980s, (b) 1990s, and (c) 2000s; and for ER-negative disease diagnosed in the (d) 1980s, (e) 1990s, and (f) 2000s.**


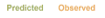


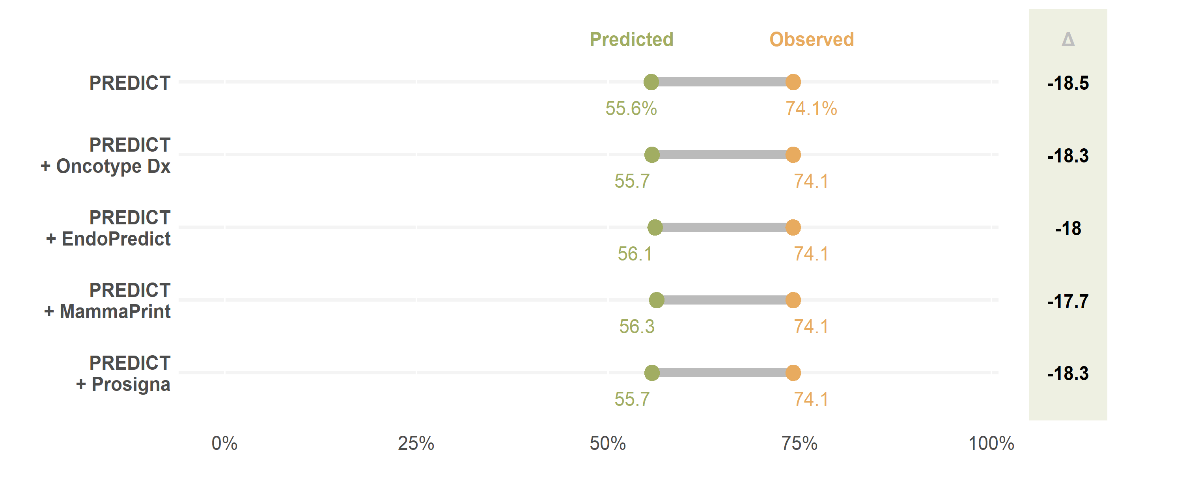

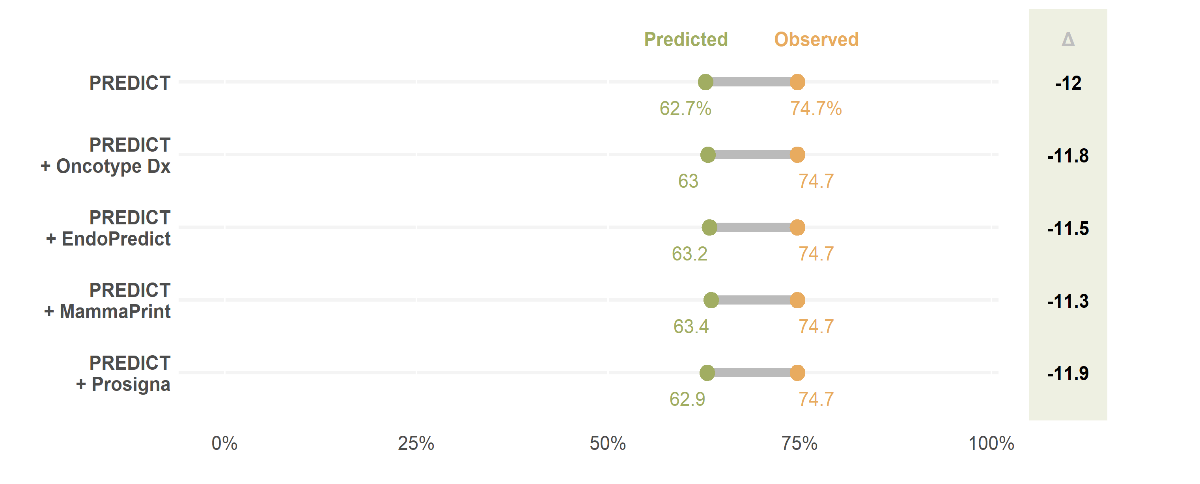

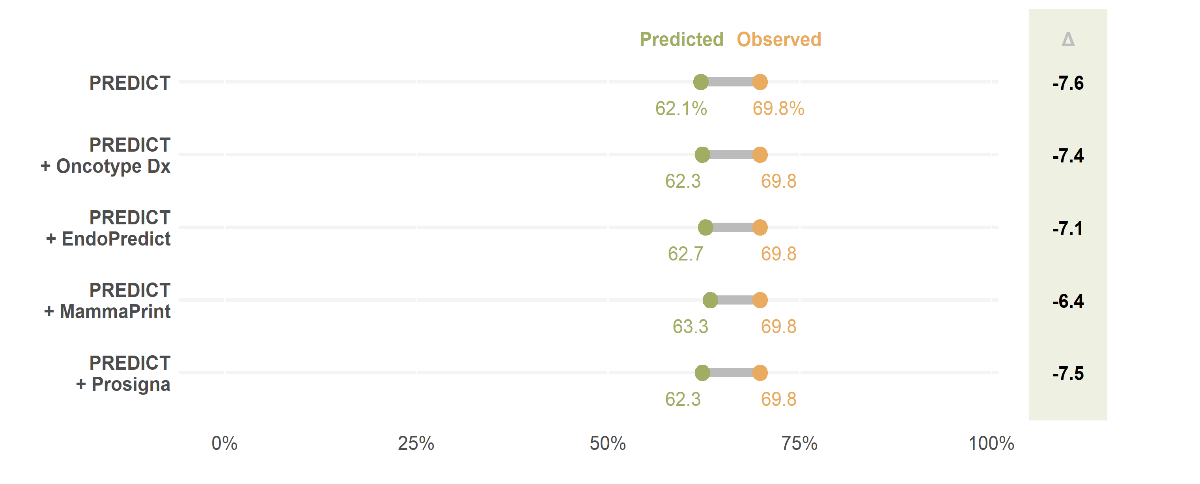


**(a)**

**(b)**

**(c)**


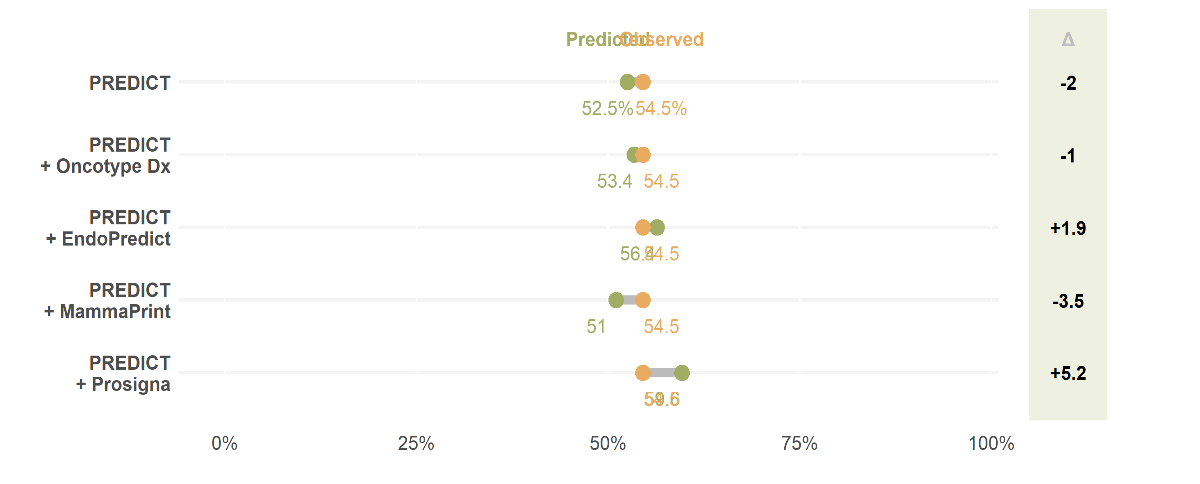


**(d)**


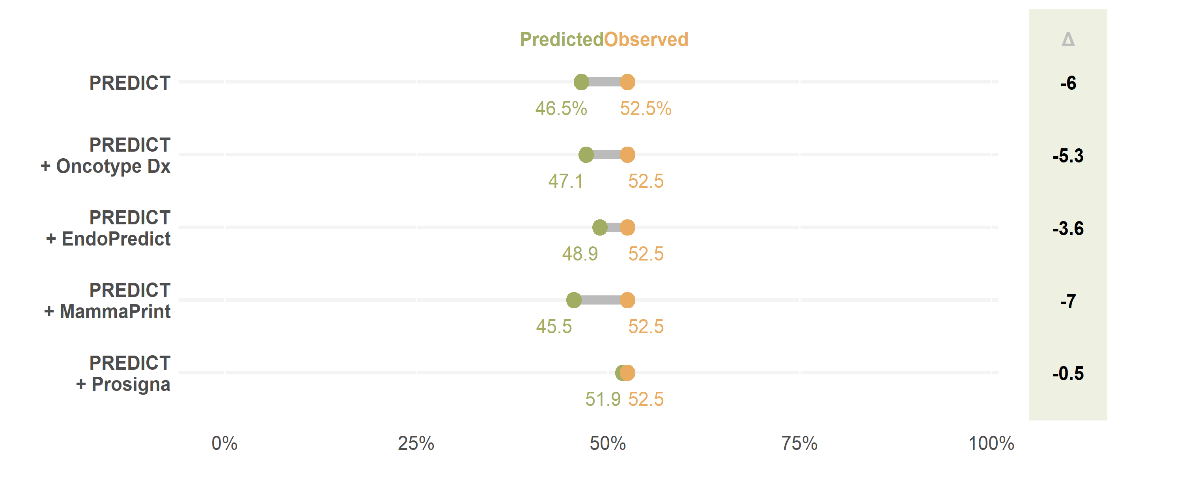

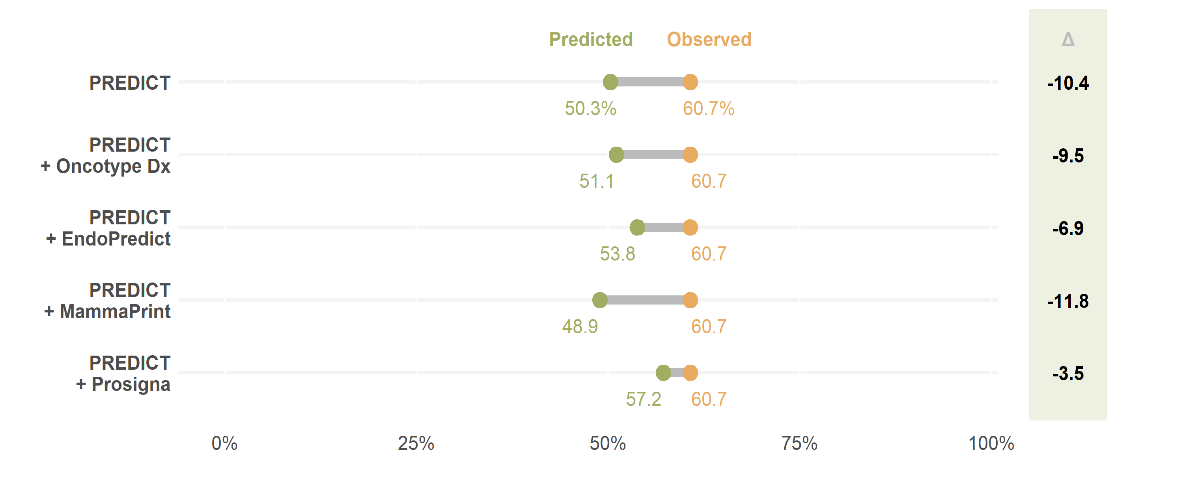


**(f)**

**(e)**


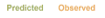


**Figure S5: 10-year breast cancer-specific survival predicted and observed patients with ER-positive disease who make up the METABRIC population: (a) cohort 1, (b) cohort 2, (c) cohort 3, (d) cohort 4, (e) cohort 5.**


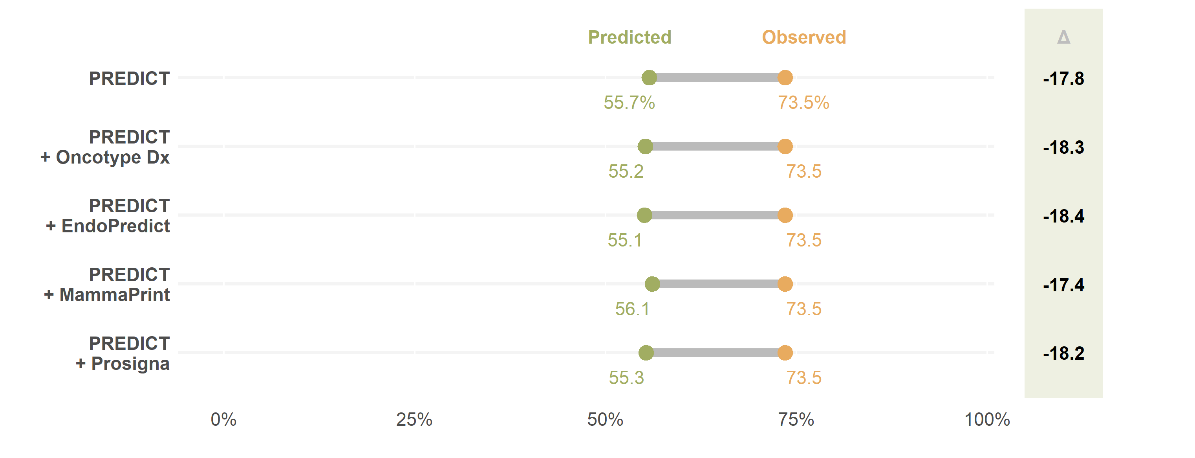

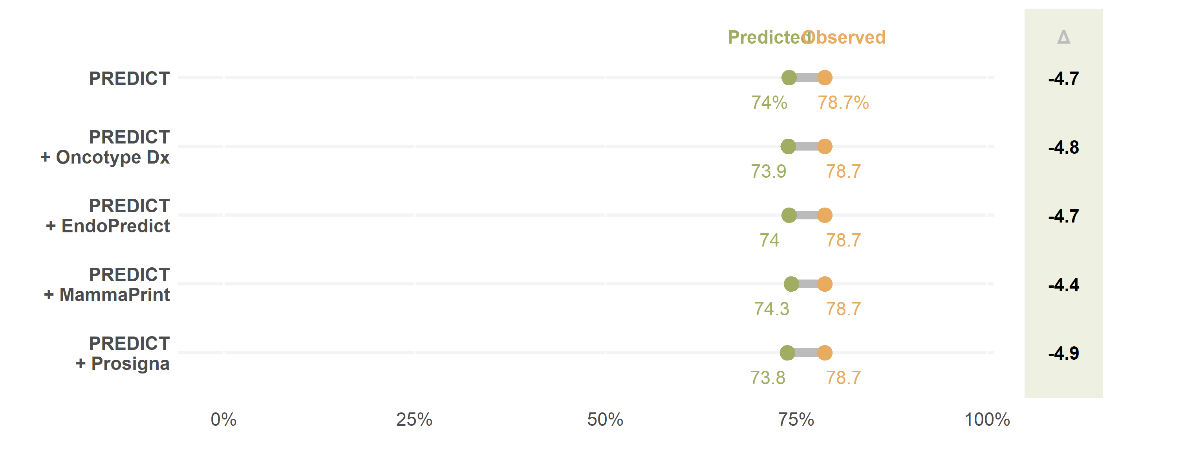

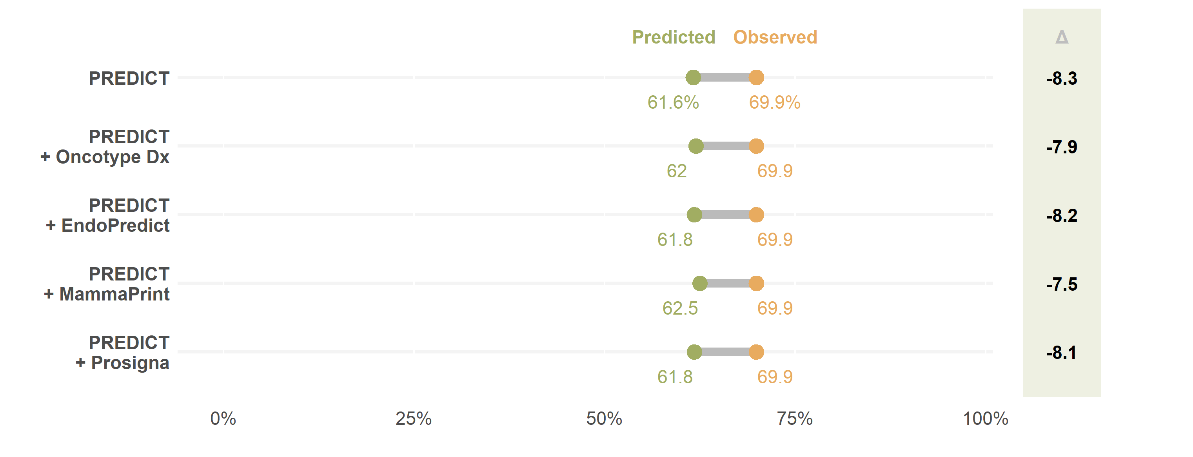

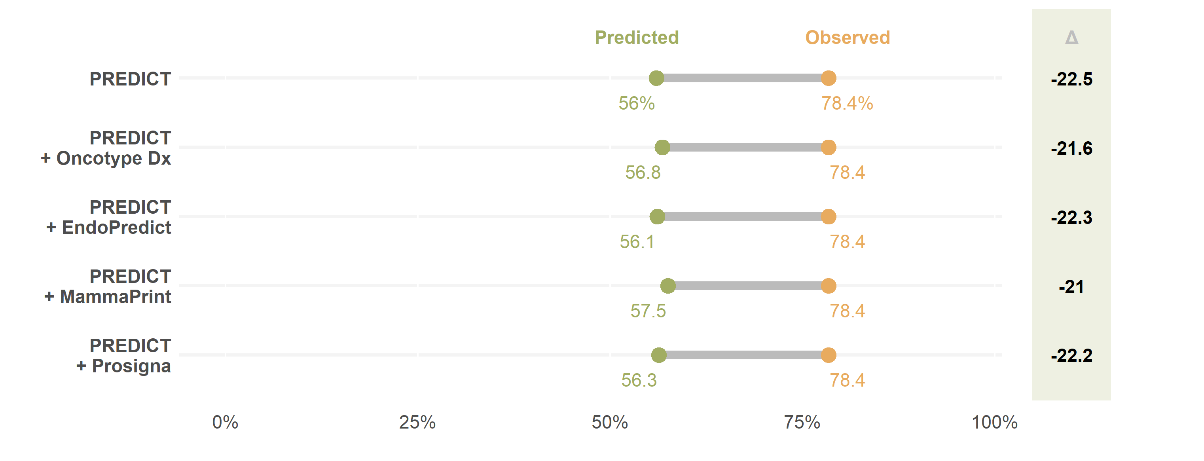

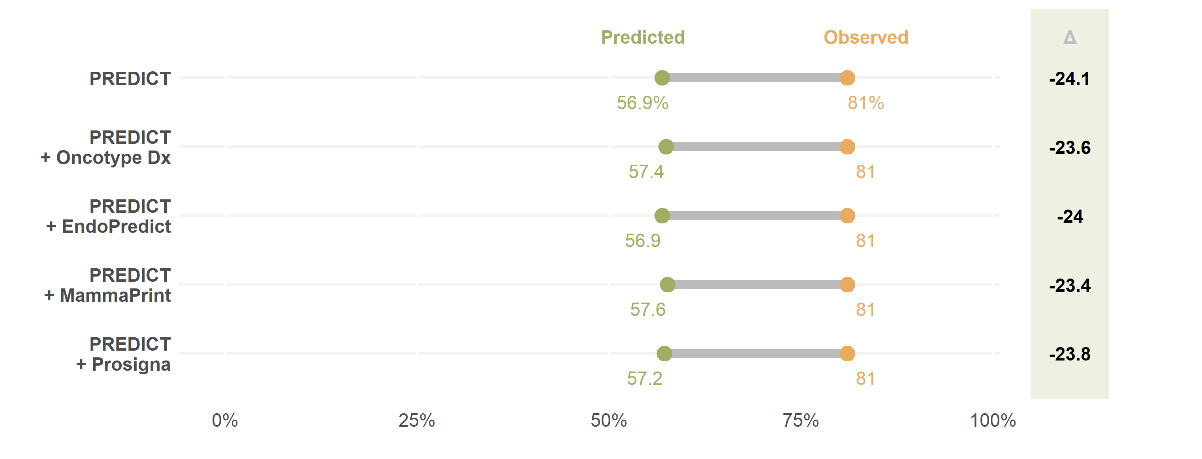


**(a)**

**(b)**

**(c)**

**(d)**

**(e)**


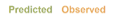


**Figure S6: 10-year breast cancer-specific survival predicted and observed patients with ER-negative disease who make up the METABRIC population: (a) cohort 1, (b) cohort 2, (c) cohort 3, (d) cohort 4, (e) cohort 5.**

**(e)**

**(d)**

**(c)**

**(b)**

**(a)**


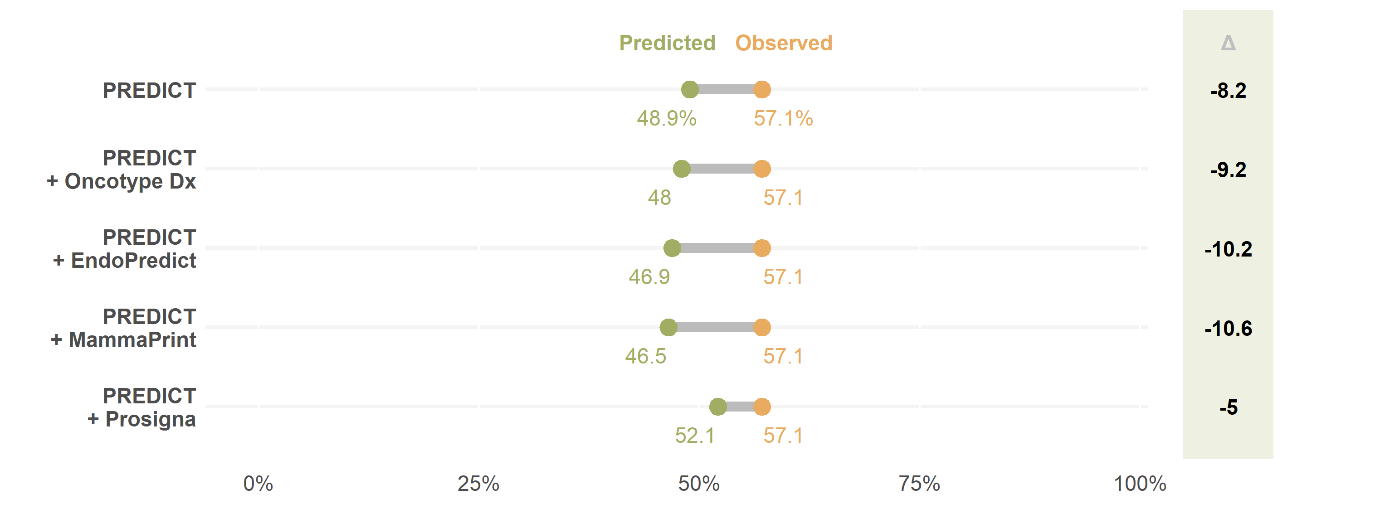

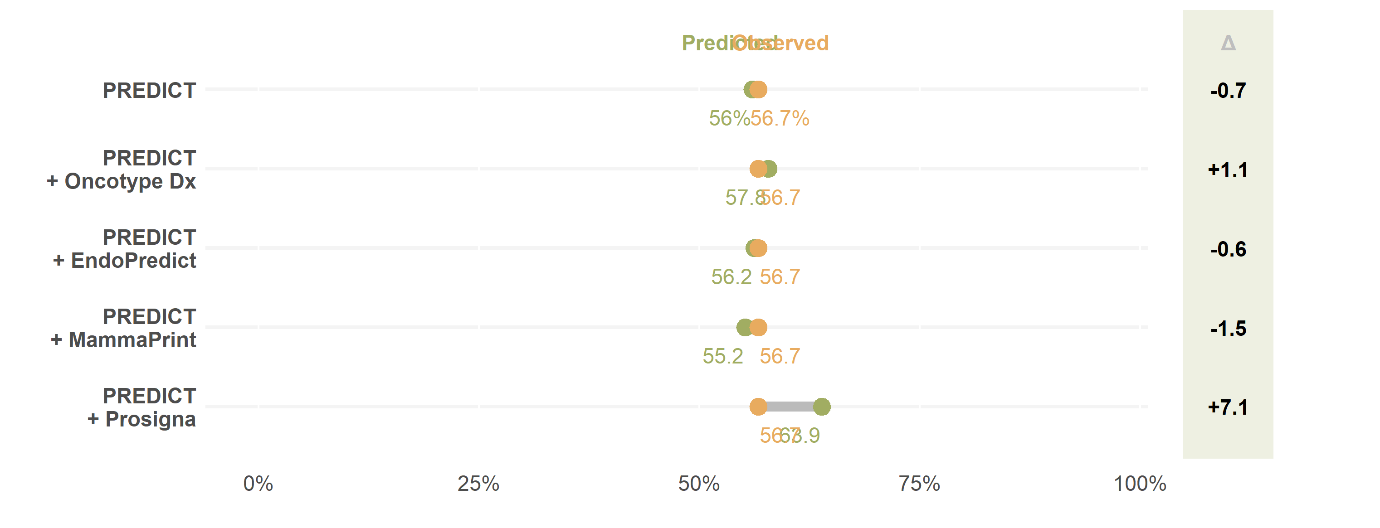

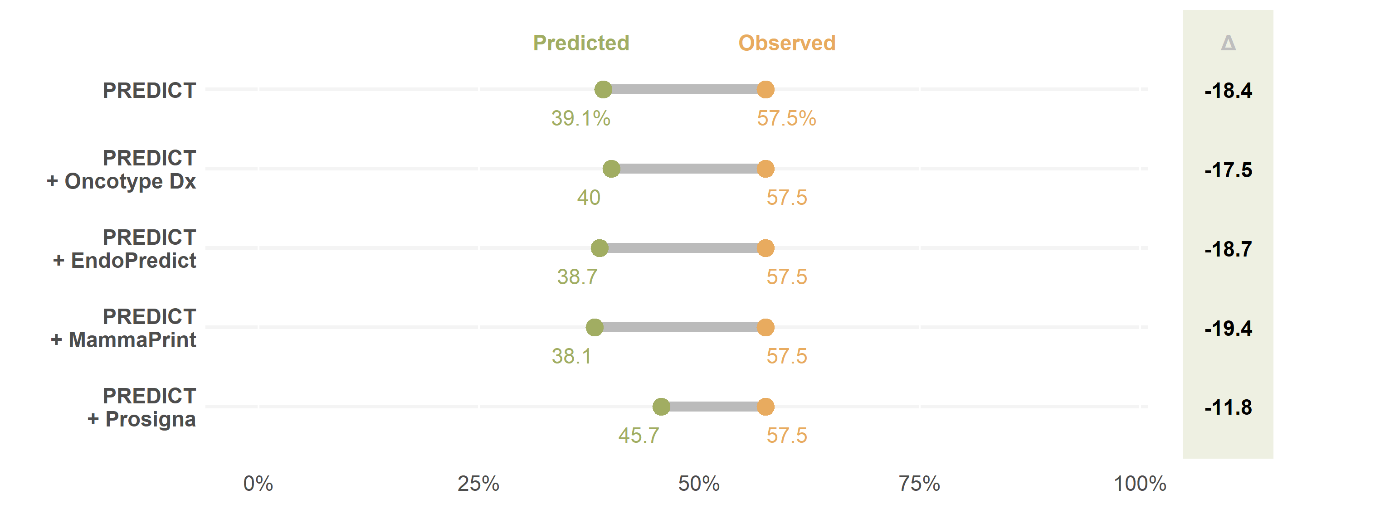

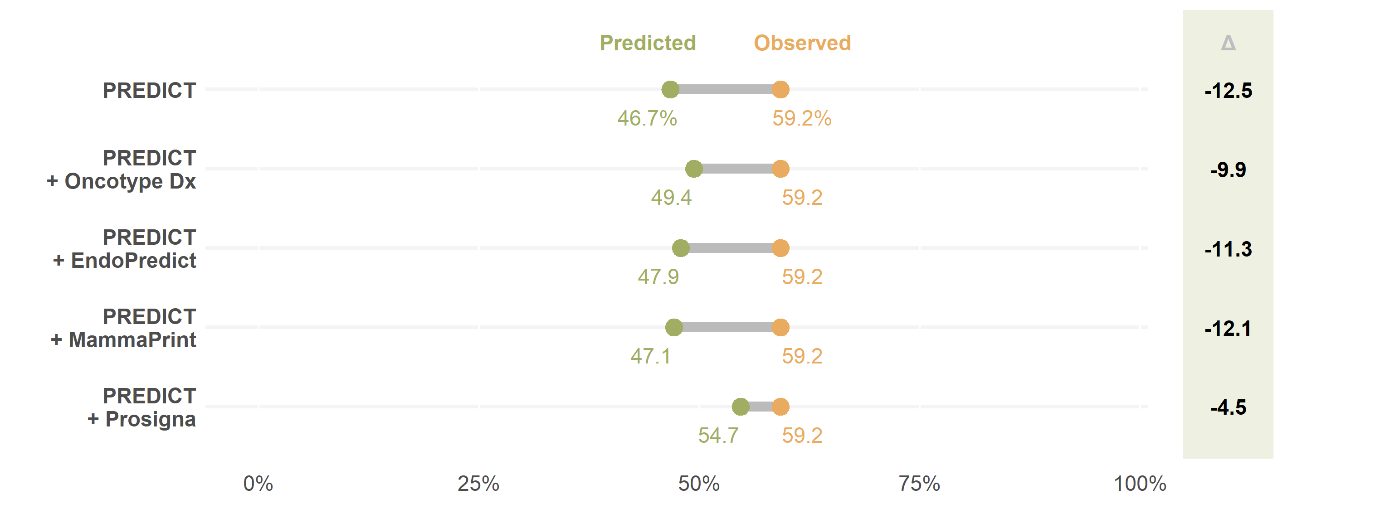

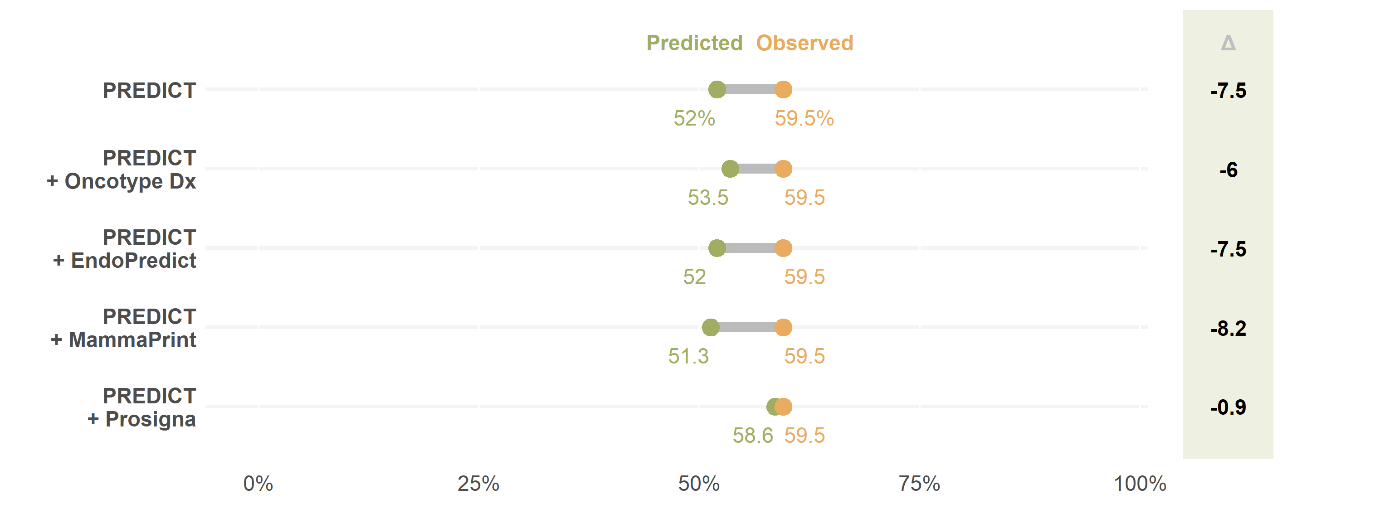

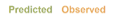

Supplement: Supplementary file 2 — Additional file 2. Supplementary Tables and Figures. [file 13058_2023_1612_MOESM2_ESM.docx]
